# Supplementary material for: Development and Characterization of a Luciferase Labeled, Syngeneic Murine Model of Ovarian Cancer
Source: Cancers (Basel). 2022 Aug 30;14(17):4219. doi: 10.3390/cancers14174219 (PMC9454869; doi:10.3390/cancers14174219)
Supplement: Supplementary file 1 [file cancers-14-04219-s001.zip › cancers-1844469-supplementary.pdf]

STOSE-luc OC Model

Figures and supplemental

Feb 2022

Russell, Lim, Peters, Wardell, Whitaker, Chang, Previs, McDonnell”

Figure S1: Generation of STOSE. M1 luc model

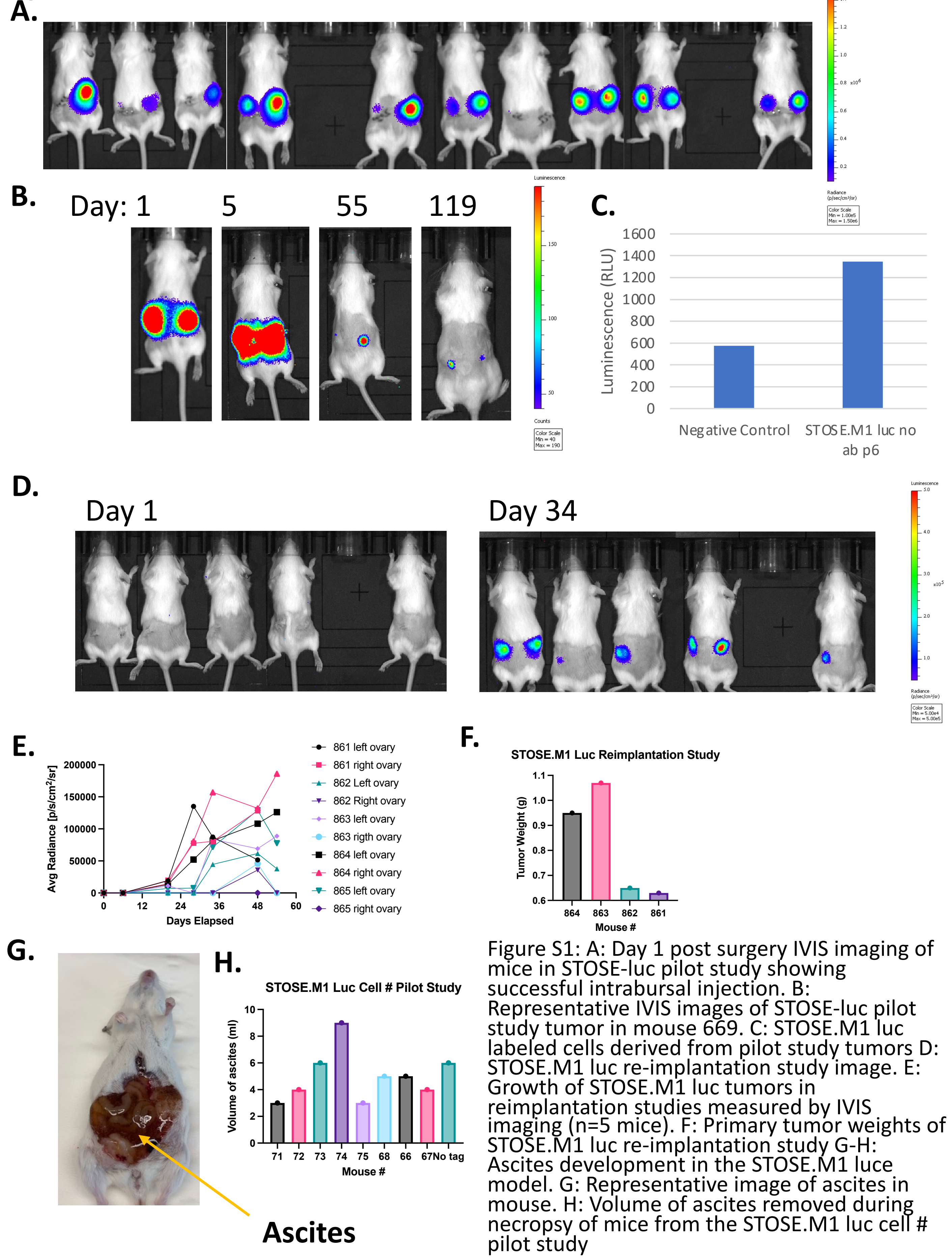

Figure S1: A: Day 1 post surgery IVIS imaging of mice in STOSE-luc pilot study showing successful intrabursal injection. B: Representative IVIS images of STOSE-luc pilot study tumor in mouse 669. C: STOSE.M1 luc labeled cells derived from pilot study tumors D: STOSE.M1 luc re-implantation study image. E: Growth of STOSE.M1 luc tumors in reimplantation studies measured by IVIS imaging (n=5 mice). F: Primary tumor weights of STOSE.M1 luc re-implantation study G-H: Ascites development in the STOSE.M1 luce model. G: Representative image of ascites in mouse. H: Volume of ascites removed during necropsy of mice from the STOSE.M1 luc cell # pilot study

Figure S2: Images of mice from the STOSE.M1 luc re-implantation study

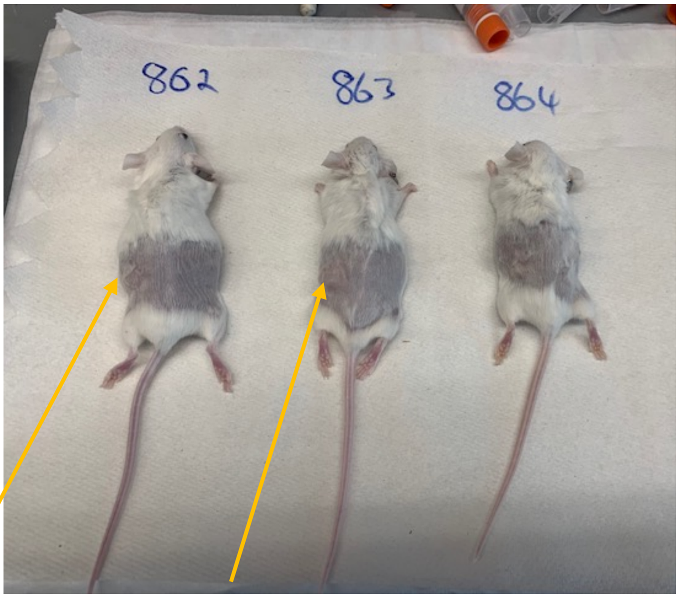

Ascites

Primary Ovarian Tumor

Metastasis

Primary Ovarian Tumor

Primary Ovarian Tumor

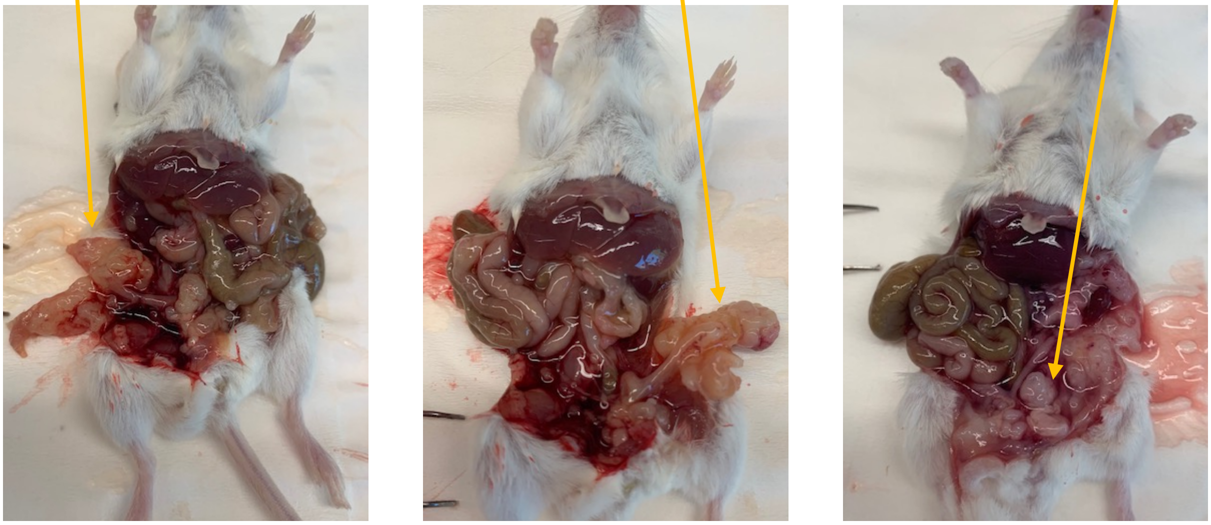

Figure S3: FlowSOM analysis of lymphoid markers on CD45+ immune populations

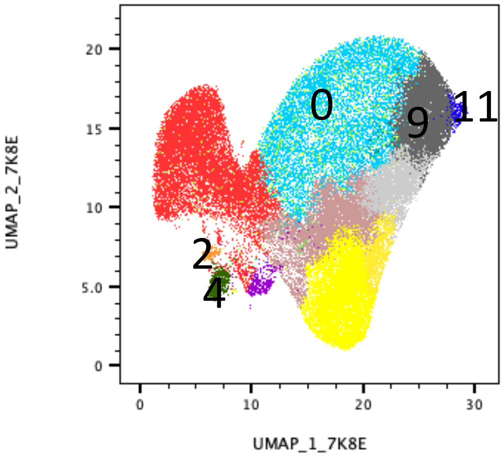

Sample Name	Subset Name	Count
concat_allsites_1.fcs	FlowSOM.Pop12	1255
concat_allsites_1.fcs	FlowSOM.Pop11	272
concat_allsites_1.fcs	FlowSOM.Pop10	3825
concat_allsites_1.fcs	FlowSOM.Pop9	8558
concat_allsites_1.fcs	FlowSOM.Pop8	8818
concat_allsites_1.fcs	FlowSOM.Pop7	103
concat_allsites_1.fcs	FlowSOM.Pop6	381
concat_allsites_1.fcs	FlowSOM.Pop5	7571
concat_allsites_1.fcs	FlowSOM.Pop4	585
concat_allsites_1.fcs	FlowSOM.Pop3	112
concat_allsites_1.fcs	FlowSOM.Pop2	284
concat_allsites_1.fcs	FlowSOM.Pop1	22677
concat_allsites_1.fcs	FlowSOM.Pop0	40559
concat_allsites_1.fcs	Primary	95000

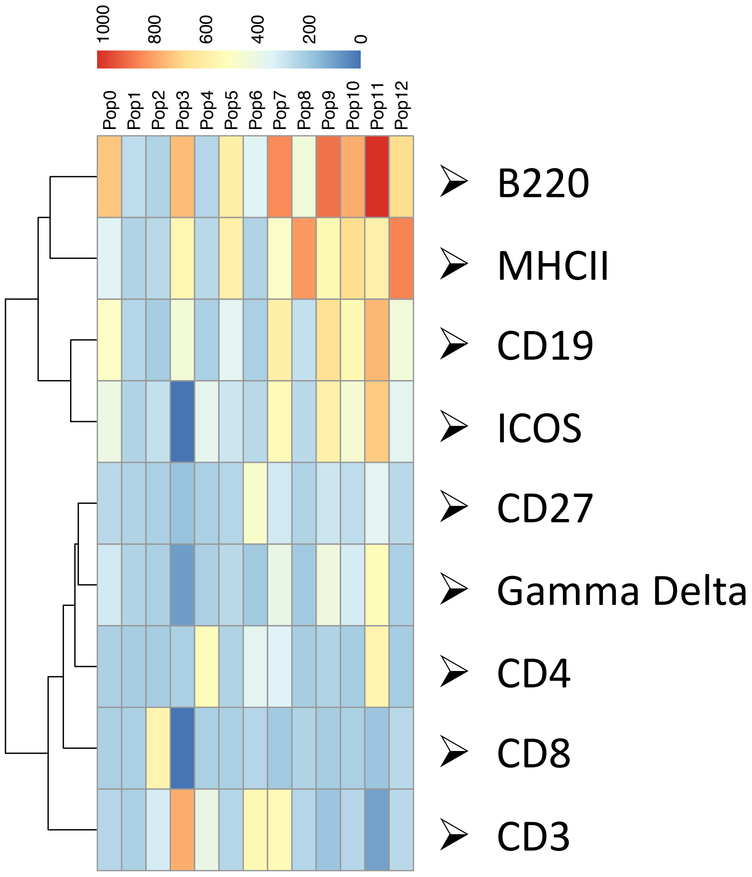

Figure S4: Immune infiltration in intrabursal model sites

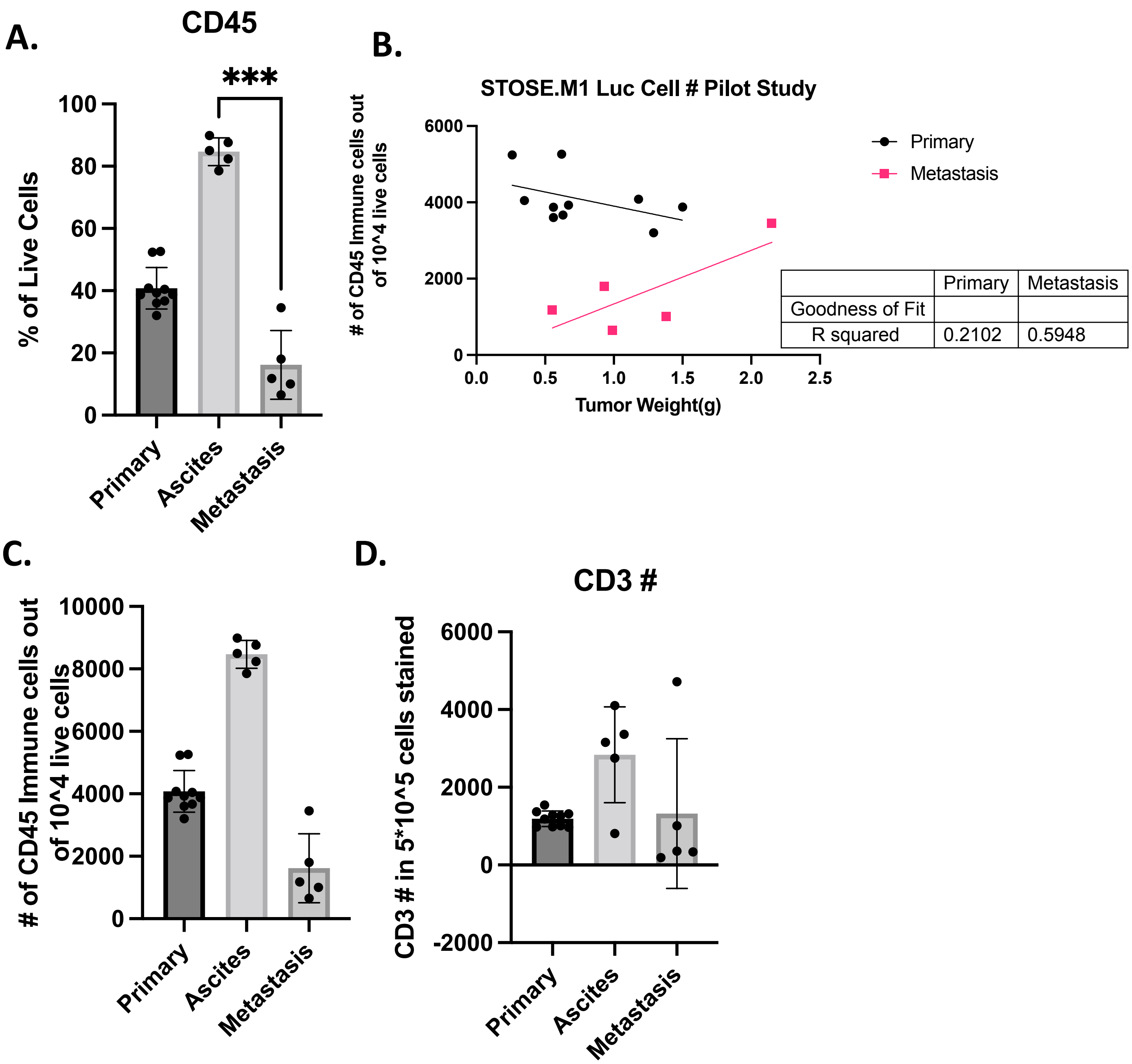

Figure S4. Immune infiltration in intrabursal model sites of STOSE.M1 luc. A: CD45 infiltration as a % of live cells. B: Number of CD45 cells normalized to live cells in primary and metastatic tumors vs weight of tumor. C: Number of CD45 cells normalized to live cells in each site. D: Absolute number of CD3 measured from 5x10^5 cells stained for flow cytometry analysis. *** $p < 0.001$

Figure S5: Representative Lymphoid Panel Gating Strategy used to assess primary tumor, ascites and metastases STOSE.M1 luc samples

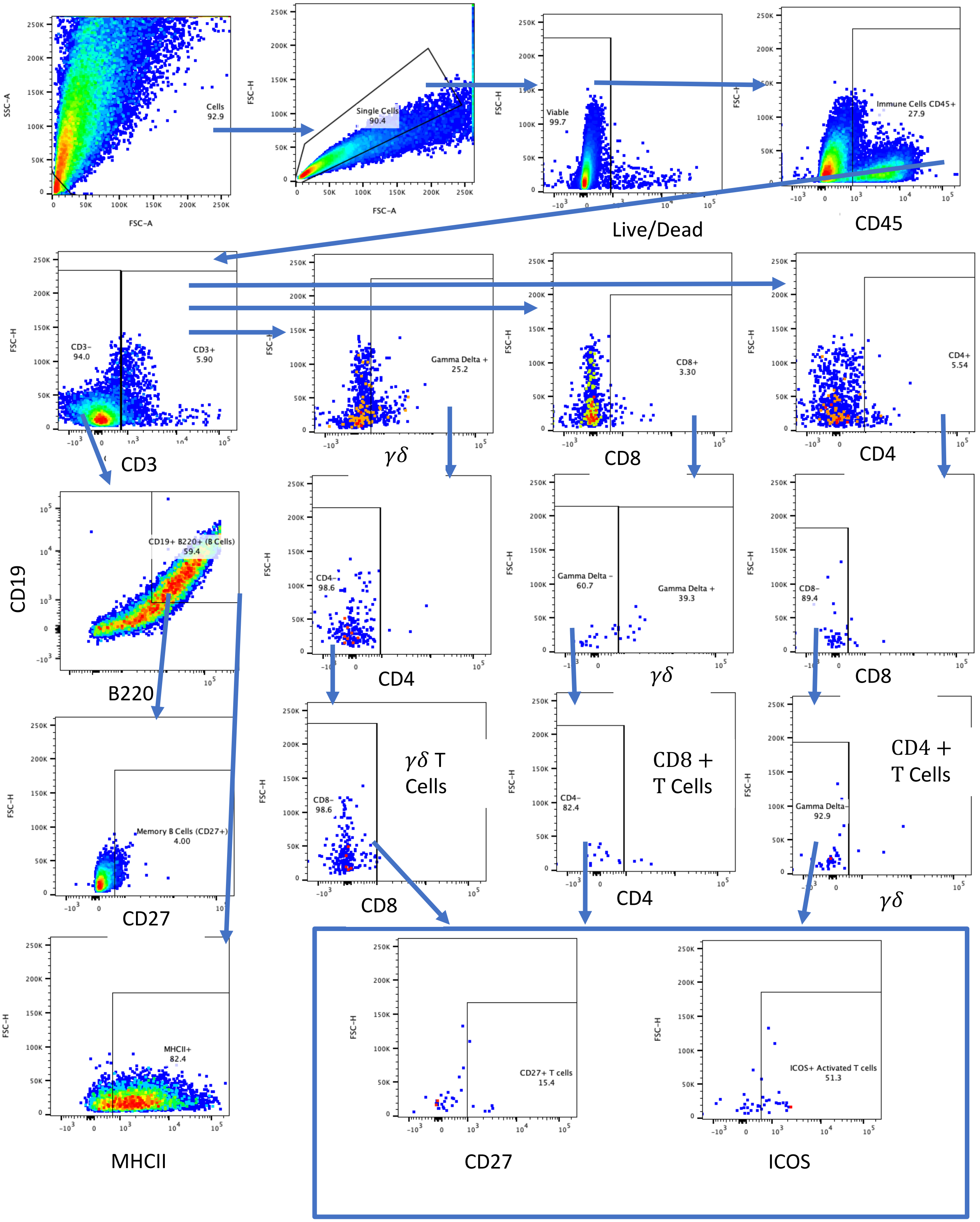

Figure S6: Expression of T cell activation markers CD44 and CD69

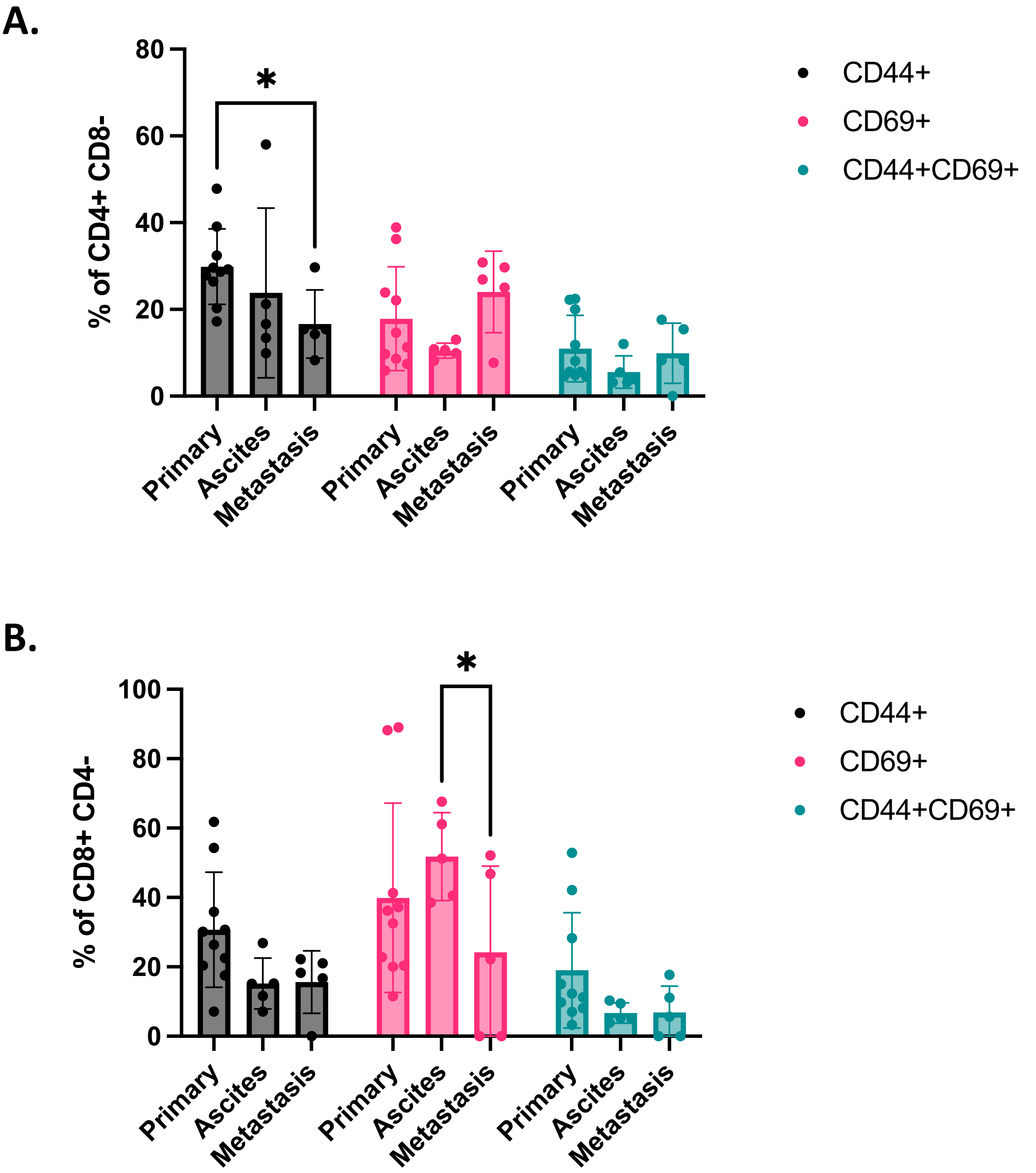

Figure S6. Expression of T cell activation markers CD44 and CD69 A: % of CD4+CD8- T cells B: % of CD8+CD4- T cells*p<0.05

Figure S7: Representative T Helper Panel Gating Strategy used to assess primary tumor, ascites and metastases STOSE.M1 luc samples

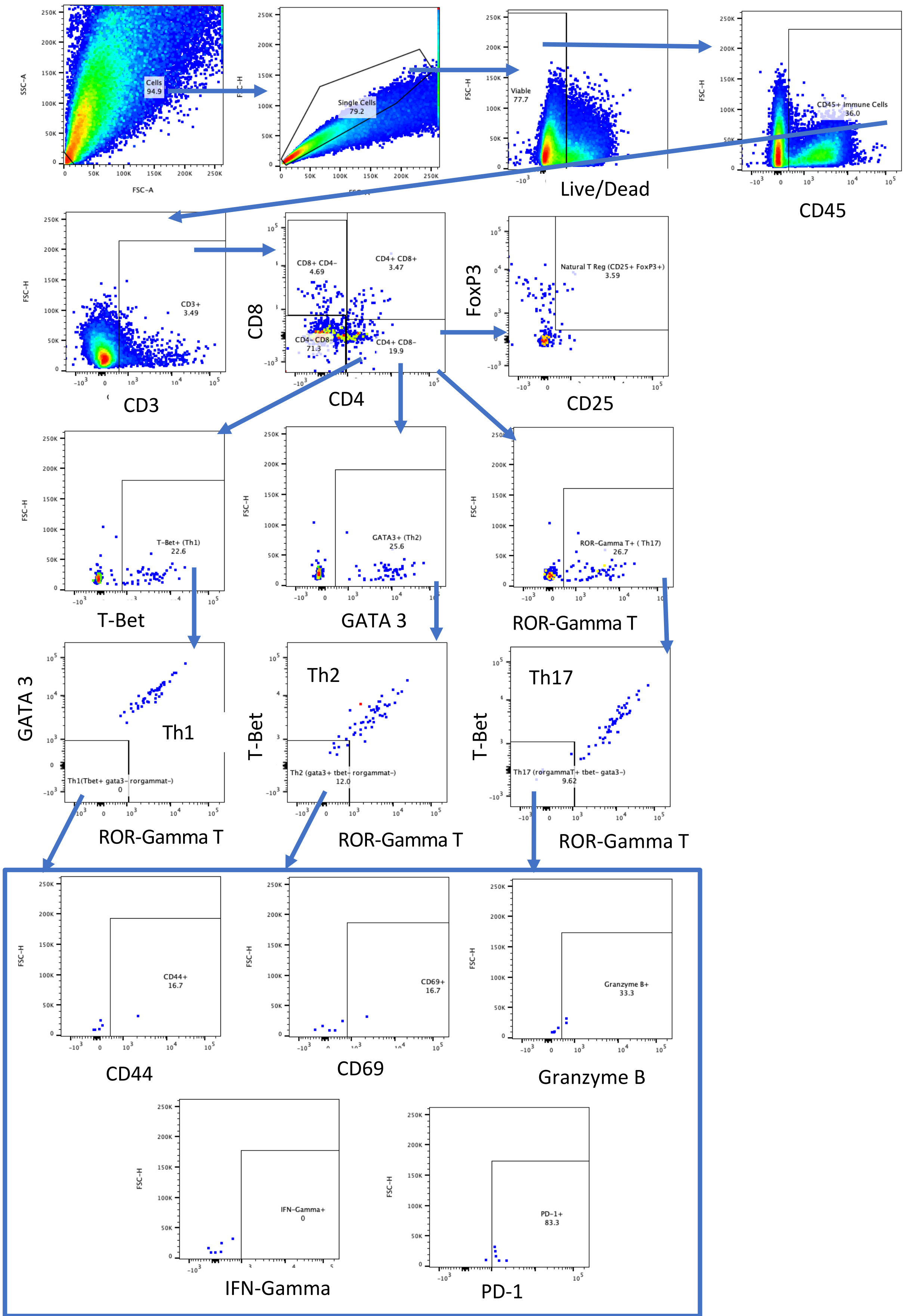

Figure S8: Representative T Memory Panel Gating Strategy used to assess primary tumor, ascites and metastases STOSE.M1 luc samples

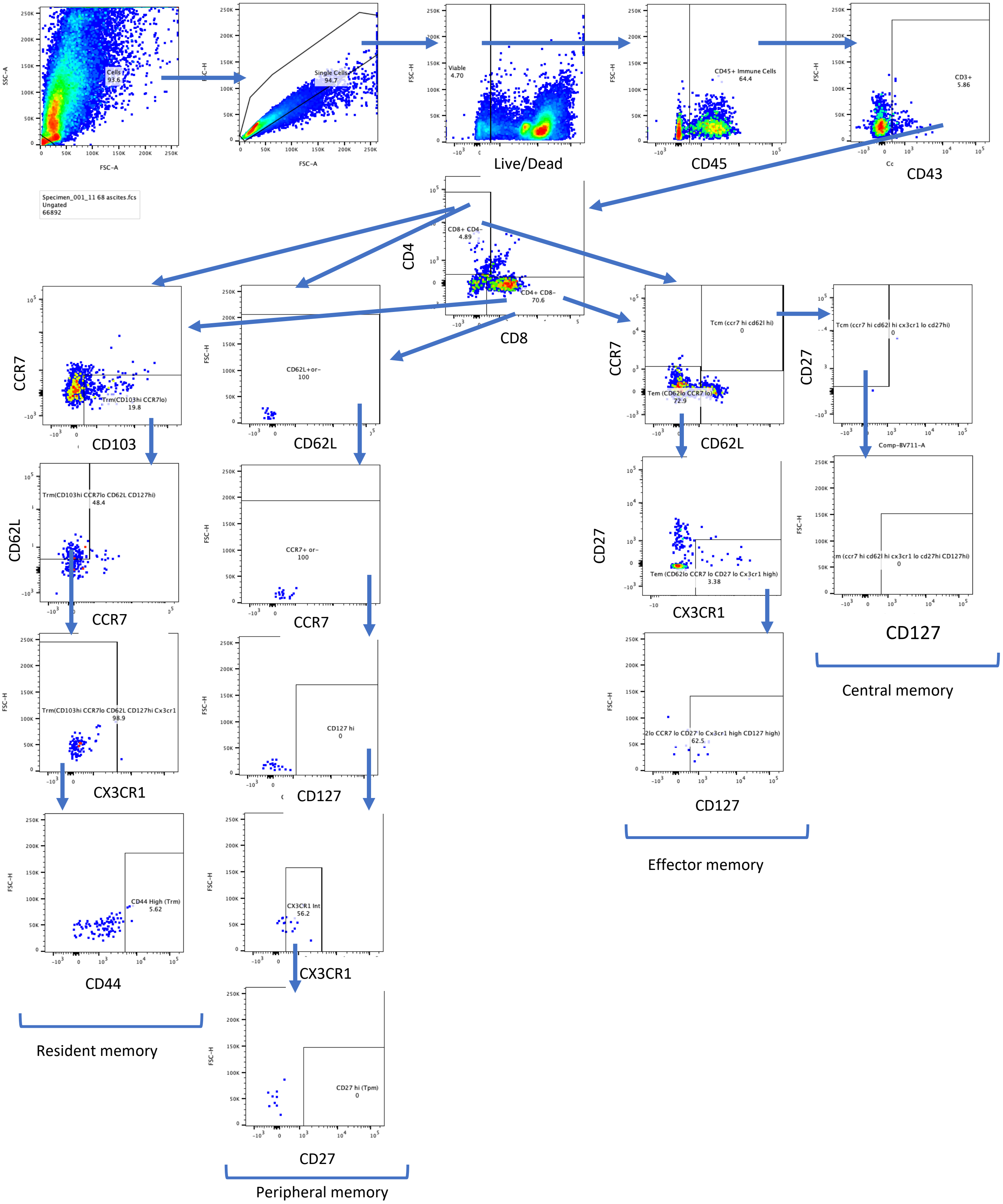

Figure S9: FlowSOM analysis of myeloid markers on CD45+ immune populations

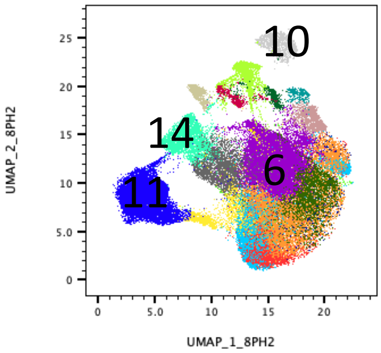

	Sample Name	Subset Name	Count
	concat_combinedsite_1.fcs	FlowSOM.Pop17	399
	concat_combinedsite_1.fcs	FlowSOM.Pop16	759
	concat_combinedsite_1.fcs	FlowSOM.Pop15	431
	concat_combinedsite_1.fcs	FlowSOM.Pop14	3061
	concat_combinedsite_1.fcs	FlowSOM.Pop13	2129
	concat_combinedsite_1.fcs	FlowSOM.Pop12	1445
	concat_combinedsite_1.fcs	FlowSOM.Pop11	10645
	concat_combinedsite_1.fcs	FlowSOM.Pop10	1381
	concat_combinedsite_1.fcs	FlowSOM.Pop9	3116
	concat_combinedsite_1.fcs	FlowSOM.Pop8	108
	concat_combinedsite_1.fcs	FlowSOM.Pop7	306
	concat_combinedsite_1.fcs	FlowSOM.Pop6	10785
	concat_combinedsite_1.fcs	FlowSOM.Pop5	1462
	concat_combinedsite_1.fcs	FlowSOM.Pop4	3239
	concat_combinedsite_1.fcs	FlowSOM.Pop3	117
	concat_combinedsite_1.fcs	FlowSOM.Pop2	40845
	concat_combinedsite_1.fcs	FlowSOM.Pop1	1681
	concat_combinedsite_1.fcs	FlowSOM.Pop0	11091
	concat_combinedsite_1.fcs	Primary	93000

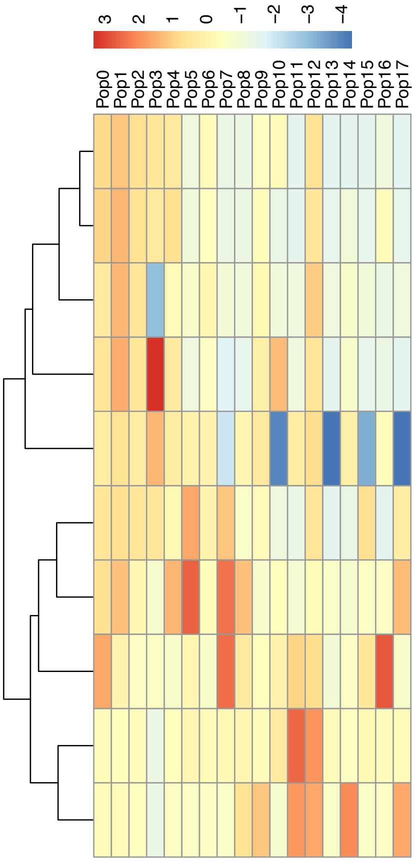

- CD206
- F480
- CD80
- CD64
- CD11b
- MHCII
- CD11c
- CD24
- Ly6G
- Ly6c

Figure S10: Representative Myeloid Panel Gating Strategy used to assess primary tumor, ascites and metastases STOSE.M1 luc samples

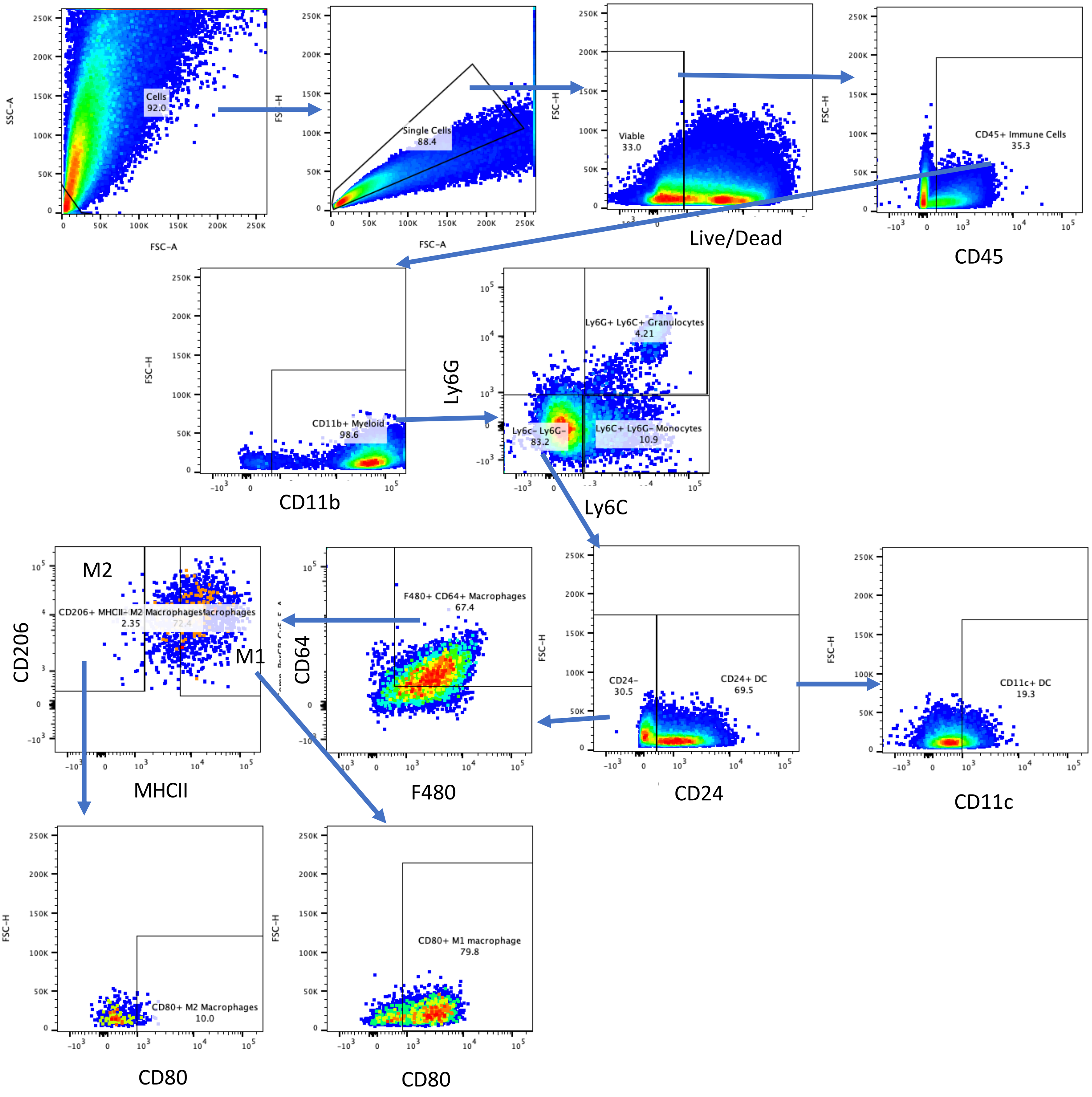

Figure S11: Representative DC Panel Gating Strategy used to assess primary tumor, ascites and metastases STOSE.M1 luc samples

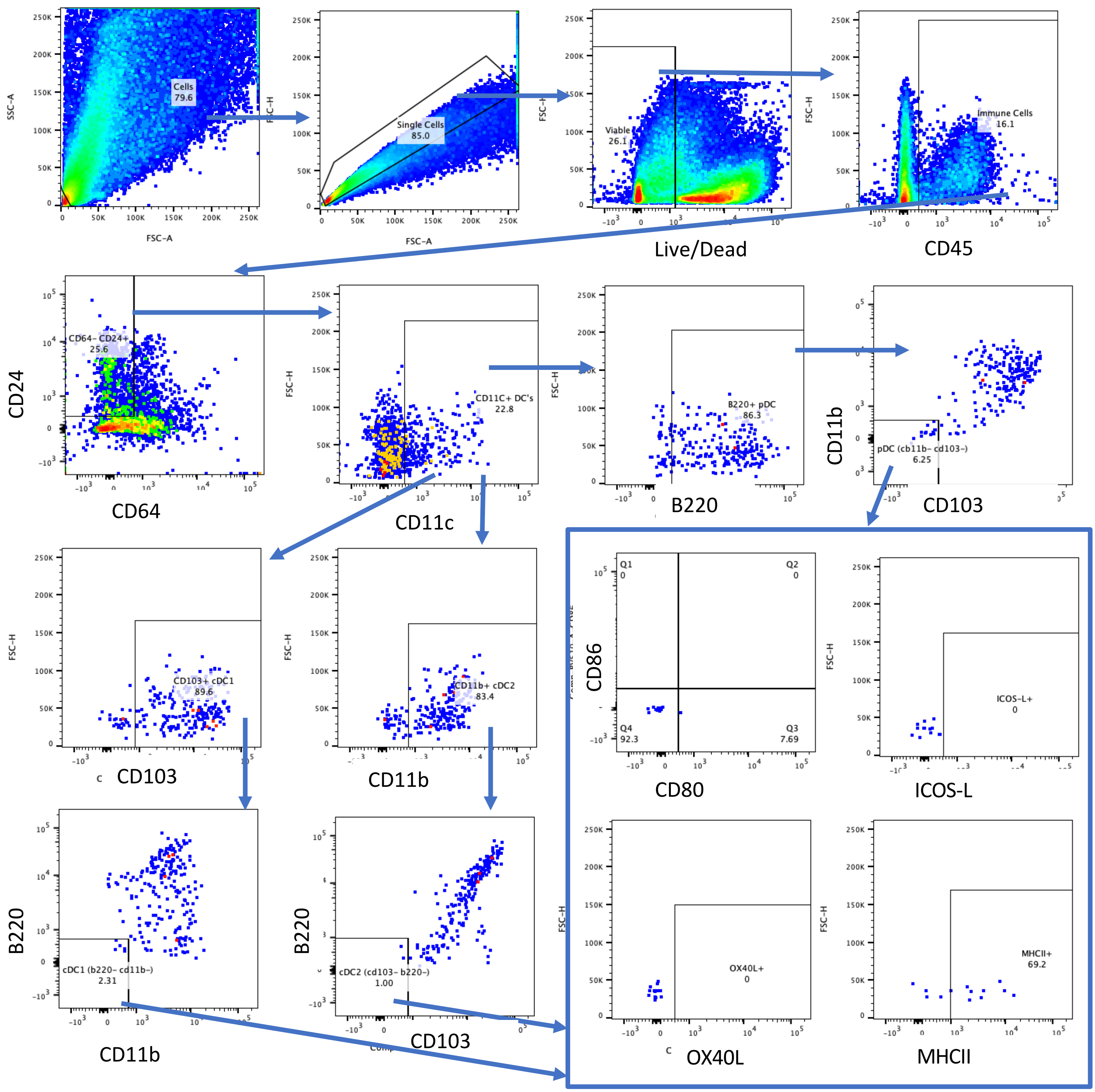

Table S1: Antibodies used in this paper

Marker	Concentration	Company	Catalogue Number	Clone
**Lymphoid Panel**				
CD27 – FITC	1:50	eBioscience	11027181	LG.7F9
CD3 – PerCP-Cy5.5	1:50	BD	560572	17A2
γδ TCR - APC	1:100	Bioleged	118116	GL3
CD45- BV605	1:800	BioLegend	103139	30-F11
CD8a- BV650	1:100	Biolegend	100741	53-6.7
MHCII – BV711	1:100	BD	563414	M5/114.15.2
CD4 – BV786	1:100	Biolegend	100551	RM4-5
CD19 – PE-TR	1:200	Invitrogen	RM7717	6D5
ICOS – Pe-Cy5	1:200	Biolegend	107708	15F9
B220 – PE-Cy7	1:100	Biolegend	103221	RA3-6B2
Live/Dead Violet - BV421	1:200	Invitrogen	L34964	
**Myeloid Panel**				
CD206 - FITC	1:100	Biolegend	141710	C068C2
CD64 – PerCP-Cy5.5	1:100	Biolegend	139308	X54-5/7.1
F480 – APC	1:100	Biolegend	123116	BM8
CD11c – APC- Cy7	1:50	BD	561241	HL3
CD24 – BUV496	1:100	BD	612953	M1/69
CD45 – BV605	1:800	BioLegend	103139	30-F11
CD80 – BV650	1:100	Biolegend	104731	16-10A1
Ly6C – BV711	1:100	Biolegend	128037	HK1.4
Ly6G – BV786	1:100	Biolegend	127645	1A8
CD11b - PE	1:100	Biolegend	101207	M1/70
MHCII – Pe-Cy7	1:600	eBioscience	25-5321-80	M5/114.15.2
Live/Dead – BV421	1:200	Invitrogen	L34964	
**T Helper Panel**				
CD3 – PerCP-Cy5.5	1:100	BD	560572	17A2
CD25- APC	1:100	eBioscience	17-0251-82	PC61.5
CD44 – AF700	1:100	Biolegend	103026	IM7
CD69 – APC-Cy7	1:100	Biolegend	104526	H1.2F3
PD-1 – BV510	1:100	Biolegend	135241	29F.1A12
CD45 – BV605	1:800	BioLegend	103139	30-F11
CD8a – BV650	1:100	Biolegend	100741	53-6.7
CD4 – BV786	1:100	Biolegend	100551	RM4-5
Live/Dead – BUV496	1:200	Thermo	L34961	
GATA3 - FITC	1:100	Thermo	53-9966-41	TWAJ
T-bet – BV421	1:100	Biolegend	644815	4B10
IFN-γ – BV711	1:100	Biolegend	505835	XMG1.2
FoxP3 – PE	1:100	eBioscience	12-5773-82	FJK-16s
ROR- γT – PE-TR	1:100	Thermo	61-6981-80	B2d
Granzyme B – PE-Cy7	1:100	Biolegend	372214	QA16A02
**T Memory Panel**				
CD27 – FITC	1:100	eBioscience	11027181	LG.7F9
CD3- PerCP-Cy5.5	1:100	BD	560572	17A2
CD62L - APC	1:100	Biolegend	104412	MEL-14
CD44 – AF-700	1:100	BioLegend	103026	IM7
CD127 – APC-Cy7	1:100	Biolegend	135040	A7R34
CD25 – BV510	1:100	BioLegend	102041	PC61
CD45 – BV605	1:800	BioLegend	103139	30-F11
CD8a – BV650	1:100	Biolegend	100741	53-6.7
CXC3R1 – BV711	1:100	Biolegend	149031	SA011F11
CD4 – BV786	1:100	Biolegend	100551	RM4-5
CCR7/CD197 - PE	1:100	PE	120105	4B12
CD103 – PE-TR	1:100	Biolegend	121429	2E7
CD69 – PE-Cy7	1:100	Biolegend	104526	H1.2F3
Live/Dead – BV421	1:100	Invitrogen	L34964	
**DC Panel**				
CD64 – PerCP-Cy5.5	1:100	Biolegend	139308	X54-5/7.1
OX40L – APC	1:100	Biolegend	108812	RM134L
CD11b – AF700	1:100	BD	561241	HL3
CD11c – APC-Cy7	1:50	BD	561241	HL3
CD24 – BUV 496	1:100	BD	612953	M1/69
CD86 – BV510	1:100	BioLegend	105039	GL-1
CD45 – BV605	1:800	BioLegend	103139	30-F11
CD80 – BV650	1:100	Biolegend	104731	16-10A1
MHCII – BV711	1:100	BD	563414	M5/114.15.2
ICOS-L – PE	1:100	Biolegend	107405	B7-H2
CD103 – PE-TR	1:100	Biolegend	121429	2E7
B220 – PE-Cy7	1:100	Biolegend	103221	RA3-6B2
Live/Dead – BV421	1:200	Invitrogen	L34964	

Table S2: Immune cell markers used to gate sub-populations of immune cells

Immune Cell	Markers
T Cells	CD45+/CD3+
CD4 T Cells	CD45+/CD3+/CD4+/CD8-/γδ-
CD8 T Cells	CD45+/CD3+/CD8+/CD4-/γδ-
γδ T cells	CD45+/CD3+/γδ+/CD8-/CD4-
B Cells	CD45+/CD3-/CD19+/B220+
Memory B cells	CD45+/CD3-/CD19+/B220+/CD27+
M1 Macrophages	CD45+/CD11b+/Ly6c-/Ly6G-/CD24-/F480+/CD64+/CD206+/ MHCII(hi)
M2 Macrophages	CD45+/CD11b+/Ly6c-/Ly6G- /CD24-/F480+/CD64+/CD206+/MHCII-
Dendritic Cells (myeloid panel)	CD45+/CD11b+/Ly6c-/Ly6G-/CD24+/CD11c+
Monocytic MDSC's	CD45+/CD11b+ /Ly6C+/Ly6G-
Granulocytic MDSC's	CD45+/CD11b+/Ly6G+/Ly6C+
Dendritic cells (DC panel)	CD45+/CD64-/CD24+/CD11C+
cDC1	CD45+/CD64-/CD24+/CD11C+/CD103+ /B220-/CD11b-
cDC2	CD45+/CD64-/CD24+/CD11C+/CD11b+/CD103-/B220-
pDC	CD45+/CD64-/CD24+/CD11C+/B220+/CD11b-/CD103-
Regulatory T Cell (T Reg)	CD45+/CD3+/CD4+ CD8-/ CD25+/FoxP3+
Th2 T cells	CD45+/CD3+/CD4+/CD8-/GATA3+/T-bet-/ROR-gamma T-
Th1 T cells	CD45+ /CD3+/CD4+/CD8-/T-Bet+/ GATA3-/RORgammaT-
Th17 T cells	CD45+/CD3+/CD4+/CD8-/ROR-Gamma T+/ GATA3-/T-bet-
Effector Memory T cells	CD45+/CD3+/CD4+ or CD8+/ CCR7(lo)/CD62L(lo)/CX3CR1(hi)/CD27(lo)/CD127(hi)
Central Memory T cells	CD45+/CD3+/CD4+ or CD8+/ CCR7(hi)/CD62L(hi)/CX3CR1(lo)/CD27(hi)/CD127(hi)
Resident memory	CD45+/CD3+/CD4+ or CD8+/ CCR7(lo)/CD62L(lo)/CX3CR1(lo/int)/CD44(hi)/CD127(hi)/CD103(hi)
Peripheral Memory	CD45+/CD3+/CD4+ or CD8+/ CCR7(+/-)/CD62L(+/-)/ Cx3CR1(int)/CD27 (hi) /CD127 (hi)
